# Barriers to Trauma Care in South and Central America: a systematic review

**DOI:** 10.1007/s00590-021-03080-3

**Published:** 2021-08-14

**Authors:** Florence Kinder, Sarah Mehmood, Harry Hodgson, Peter Giannoudis, Anthony Howard

**Affiliations:** 1 LIRRM, Leeds University, Leeds, UK; 2 Leeds Teaching Hospitals NHS Trust, Leeds, UK; 3 Leeds Orthopaedic Trauma Sciences, Leeds University, Leeds, UK; 4 Leeds General Infirmary, Leeds, UK; 5 NDORMS, University of Oxford, Oxford, UK; 6 Academic T&O Unit, Clarendon Wing, D floor, Great George Street, Leeds, LS1 3EX UK

**Keywords:** South America, Trauma, Barriers, Health Care Access

## Abstract

**Introduction:**

Trauma is widespread in Central and South America and is a significant cause of morbidity and mortality. Providing high quality emergency trauma care is of great importance. Understanding the barriers to care is challenging; this systematic review aims to establish current the current challenges and barriers in providing high-quality trauma care within the 21 countries in the region.

**Methods:**

OVID Medline, Embase, EBM reviews and Global Health databases were systematically searched in October 2020. Records were screened by two independent researchers. Data were extracted according to a predetermined proforma. Studies of any type, published in the preceding decade were included, excluding grey literature and non-English records. Trauma was defined as blunt or penetrating injury from an external force. Studies were individually critically appraised and assessed for bias using the RTI item bank.

**Results:**

57 records met the inclusion criteria. 20 countries were covered at least once. Nine key barriers were identified: training (37/57), resources and equipment (33/57), protocols (29/57), staffing (17/57), transport and logistics (16/57), finance (15/57), socio-cultural (13/57), capacity (9/57), public education (4/57).

**Conclusion:**

Nine key barriers negatively impact on the provision of high-quality trauma care and highlight potential areas for improving care in Central & South America. Many countries in the region, along with rural areas, are under-represented by the current literature and future research is urgently required to assess barriers to trauma management in these countries.

No funding was received. *Clinical Trial Registration*: PROSPERO CRD42020220380.

## Background

Trauma, defined as serious injury to the body (blunt force or penetrating) [1], presents a significant concern in Central and South America. Traumatic injury is common, and is significant cause of mortality, particularly in the young [2]. Access to safe, timely and affordable care is vital. The lack of access to emergency trauma care is a significant public health issue, particularly in densely populated countries such as Mexico and Brazil, where trauma is the biggest killer in children and young adults [3, 4].

Further, trauma accounts for high proportions of disabilities. In Mexico, almost two thirds of disabilities and half of deaths in 16–45 year olds are caused by unintentional trauma [2, 5]**.** This is particularly significant as this age range encompasses most of the working population, hence improving mortality and morbidity rates is projected to provide economic benefit to low-to-middle-income countries (LMIC) [5]**.** It has been estimated that reducing mortality rates in LMIC to those seen in higher-income countries (HIC) would lead to a $760 billion saving and save two million lives each year [6]**.**

South and Central America encompasses 21 countries (Argentina, Bolivia, Brazil, Chile, Colombia, Ecuador, Falkland Islands, French Guiana, Guyana, Paraguay, Peru, Suriname, Uruguay, Venezuela and El Salvador, Costa Rica, Belize, Guatemala, Honduras, Nicaragua and Panama). For the purpose of this review, the Falkland Islands and French Guiana have been excluded on the grounds of being territories of other countries.

The region is economically diverse; according to the World Bank, it consists of three high-income countries (HIC), 12 upper middle-income countries (UMICs), four lower middle-income countries (LMICs) and no low-income countries (LIC) [7]. While only a small number of countries in the region fall into the LMIC category, disparities in availability and quality of trauma care account for approximately two million preventable deaths in LMIC and LICs, annually [8, 9]. It should also be noted that almost one third of the region live in poverty (184 million) [10]. Further, there is great inequality within the region; as a whole, Latin America alongside Sub-Saharan Africa is the most unequal region of the world and has some of the most ingrained health inequalities [10, 11]. Research analysing the trauma systems in Mexico and Brazil found substantial differences in the quality of trauma care between large cities, and that of smaller cities and more rural environments, for example lack of staff with ATLS (Advanced Trauma Life Support) training at smaller rural centres. [2]. It is further highlighted the lack of injury preventative measures in rural Latin America as well as concerns about capabilities of transport vehicles for rural issues with many ambulance services lacking basic monitoring and paramedic care [12]. Healthcare systems in South and Central America encompass a mix of state and private services. Health expenditure as a proportion of GDP is low. Publicly funded care typically receives less than 6% of GDP, with rankings of poor–moderate for attainment of universal coverage for most [13].

Gold standard trauma care is considered to be that provided by Level 1 Trauma Centres (defined as somewhere able to provide ‘definitive care for every aspect of injury’ [14]). It is highlighted that grading systems vary, for example, in Mexico, Level 3 is the equivalent of Level 1. Top-level centres generally offer greater access to resources as well as speciality doctors. The American Trauma Society also reference commitments to prevention via public education as well as research innovation to develop trauma care. It is further noted all levels should have quality assessment systems in place [2, 15]. They are also considered to provide greater levels of training exposure and improve residency outcomes [16].

It is worth highlighting these standards when considering what barriers may be faced in achieving such levels of care.

Trauma remains one of the leading causes of death worldwide [17], accounting for 10% of the global burden of disease [18]**.** The highest demand for trauma care is seen in LMIC, which report 90% of all trauma deaths [19]. The disparity in mortality rates between LMIC and high-income countries (HIC) is projected to increase to 8% by 2030 [20]. It has been previously identified that trauma care service is an area of healthcare that is generally underdeveloped and lacks resources including medical staff and transportation [21–23]. A total of 45% of deaths and 35% of disability-adjusted life years (DALYs) could potentially be tackled by improvements to trauma care systems [23]**.** The Lancet Global Health Commission on High-Quality Health Systems [24] identifies healthcare systems in LMICs as a research priority in terms of accurate data measurements and improved assessment. It is hoped a review of current literature will provide a summary of the present understandings of barriers to trauma care in South and Central America and to provide groundings for a further Delphi analysis.

## Search methods

A systematic search of the following databases was carried out: OVID MEDLINE, OVID MEDLINE In-Process and Other Non-Indexed Citations, OVID EMBASE, EBM Reviews (Cochrane DSR, ACP Journal Club, DARE, CCA, CCTR, CMR, HTA, NHSEED) and Global Health. Kironji et al.’s strategy for ‘Identifying barriers for out of hospital emergency care in low- and low–middle-income countries: a systematic review’ [23] was used as a starting framework and expanded to streamline the search to the specific research question. The Preferred Reporting Item for Systematic reviews and Meta-Analysis (PRISMA statement) was used to guide the reporting of findings.

Trauma was defined as blunt, penetrating or serious injury from an external force, including injury from burns. Emergency medicine was considered synonymous to trauma care, and pre-hospital response to traumatic injury was also included as part of this broad definition. The search included keywords and controlled vocabulary words (MeSH terms) related to trauma care, barriers to care, and South and Central America (Appendix 1—search strategy). Boolean operators (OR and AND) were utilised to combine concepts. The search was limited to studies from the decade preceding 1 October 2020, English language (where suitable translations were available, these were included) and human subjects. Case reports and case control studies were excluded, as were conference abstracts. Review articles referring to emergency medicine outside of the pre-defined definition of ‘trauma’ for example, obstetric emergencies and sepsis, were omitted. Table 1 details the full inclusion and exclusion criteria. No funding was received for the review. Systematic review registration PROSPERO CRD42020220380.

### Assessing risk of bias

The RTI item bank for assessing bias and confounding in observational studies was used. Aspects considered included: inclusion/exclusion criteria, recruitment, comparator groups, differences to protocol, use of valid/reliable measures, differences in follow-up, missing data or outcomes and confounding. Questions on blinding of assessor and harms were omitted as not strictly relevant to the included literature.

## Results

**Fig. 1:**
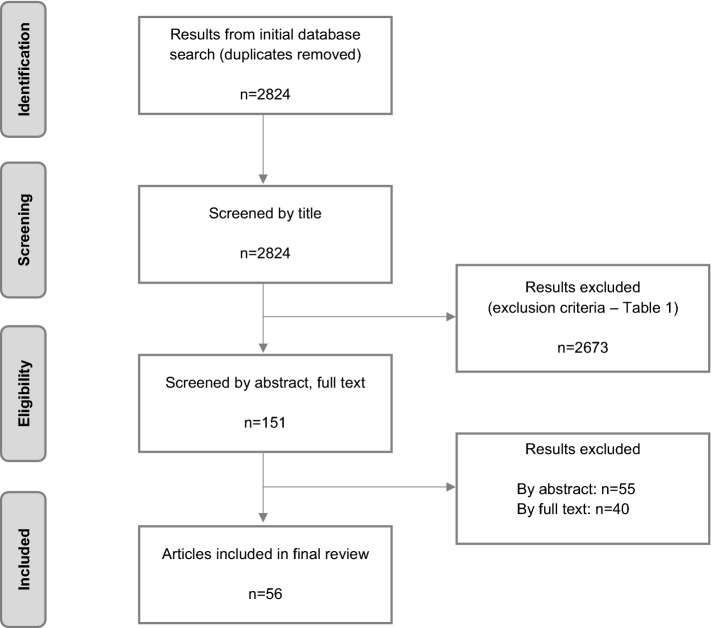
Literature search for barriers to trauma care in South and Central America; PRISMA—Preferred Reporting Items for Systematic Reviews and Meta-Analyses

**Fig. 2:**
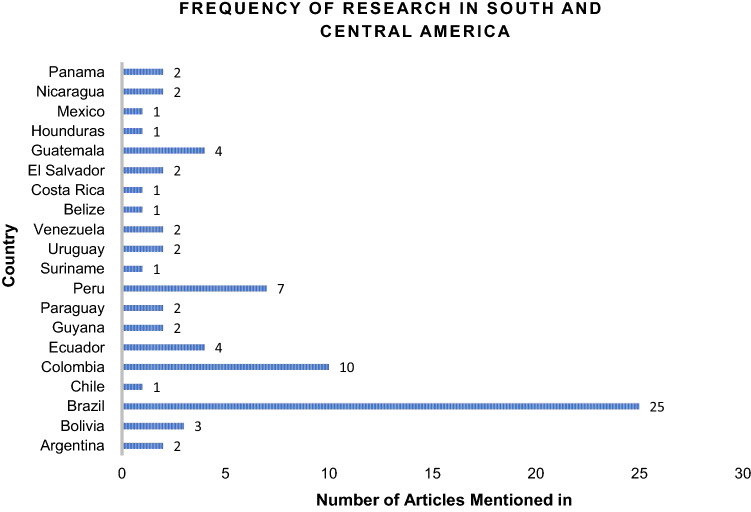
Number of times each of the 20 countries included were mentioned by articles reviewed

**Fig. 3:**
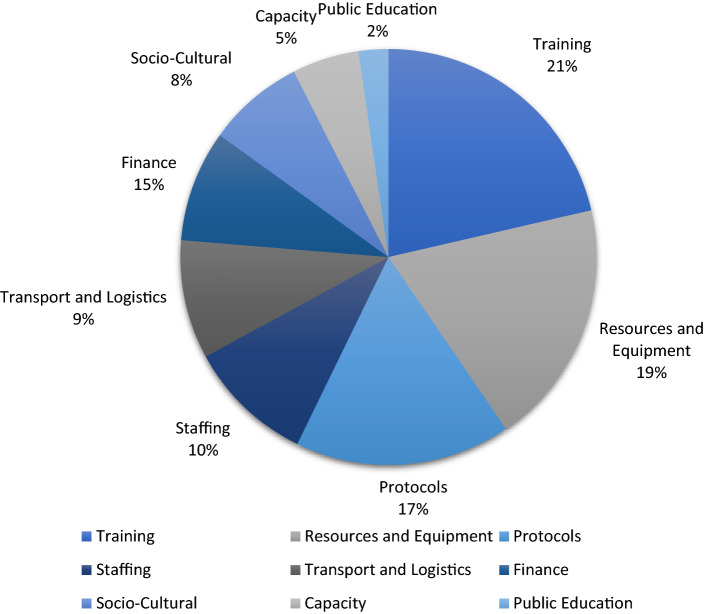
Frequency with which each of the nine broad themes identified were appeared in the articles reviewed

The search yielded 2824 results. Once duplicates were removed, 151 were selected for initial review (Fig. 1). Ninety-five did not meet inclusion criteria, leaving 56 articles for full review. Twenty countries included in the study were referenced at least once (Fig. 2). Brazil was the most referenced (25 articles). Chile, Suriname, Costa Rica, Belize, Honduras and Mexico were only mentioned once. Following full text review, nine broad themes were identified, Fig. 3 shows the frequency with which these themes arose, and they are discussed in turn below.

### Resources and equipment

A lack of resources and equipment were highlighted in 58% (33/57) of papers [9, 25–56]. This was a deficiency in both pre-hospital and hospital sectors. Substantial differences between urban and rural settings was described. Equipment including beds, diagnostic and interventional equipment including CT/MRI scanners, ventilators, catheters, and medications were also described as in short supply. In some cases, resources were in reasonable supply, but unable to cope with sharp influxes of need, such as during natural disasters. In pre-hospital care, a lack of emergency dispatch teams and ambulances were also lacking, as well as poor communication systems such as a lack of a universal emergency number. Computer systems were also described as underdeveloped with unreliable internet access.

### Staff

Understaffing across emergency care was identified in 30% (17/57) of papers [8, 21, 22, 24, 26, 30–35, 47, 49, 51–54]. A short supply of surgeons, anaesthetists, radiologists, nursing staff and paramedic staff was specifically noted. Staffing issues were enhanced by high turnover rates, as well as a tendency for inexperienced professionals with ‘general’ training being recruited for specialist roles, such as emergency dispatch.

### Training

Lack of training was referenced as a barrier to trauma care in 65% (37/57) of papers reviewed [9, 27–29, 31–36, 38, 40–46, 49, 53, 55, 57–72]. There was a lack of specialist trauma training for doctors across all grades and a lack of postgraduate training for Emergency Medicine clinicians. Six papers referenced this in context to a lack of knowledge in protocols of initial assessment and standardised approaches to care. Five papers report the lack of opportunity for continued professional development and regular training. Three papers reported a lack of audit and quality improvement, such as the absence of morbidity and mortality meetings.

### Protocol

The lack of standardised protocols and guidelines was highlighted in 51% (29/57) of papers [9, 25, 27–32, 36, 37, 40–44, 47, 52, 54, 55, 59, 61, 62, 65–67, 71–74]. Key themes relating to lack of protocol include those in relation to record-keeping (six papers) and pre-hospital communication, (six papers). Eleven papers raise lack of protocol in context of triage and assessment, leading to patients being seen solely in order of presentation rather than clinical need. On several occasions, this was linked to lack of training.

### Financial

Financial barriers were mentioned in 26% (15/57) papers [27, 31, 35, 37, 41, 46, 47, 52, 57, 60, 72, 73, 75–77]. Barriers affecting staff, patients, hospitals and general infrastructure were discussed. Four articles referred to the barriers faced in accessing care based on ability to pay. Three papers reported how finance was a barrier to training, e.g., lack of funding for ATLS, or in Guatemala, lack of funding to expand pre-hospital training. Research is also hindered by lack of finance, with two specifically referring to lack of research budgets and lack of funding to hire staff to work on such projects, e.g., data collection. In Colombia, staff are reported to switch to ICU from Emergency Medicine residencies where the financial remunerations are better.

### Transport and logistics

A total of 28% (16/57) of articles reference transport and logistical concerns as a barrier to trauma care [25, 26, 29, 30, 41, 46, 47, 49, 50, 52, 55, 56, 65, 74, 78, 79]^.^ Eight articles highlight issues with ambulances including locations of ambulance bases, time taken to deploy ambulances, time taken to reach patient, distance from hospital and traffic, all of which increase pre-hospital time. Six articles report how rural environments and mountainous terrain in areas such as Honduras, Bolivia, Brazil, Suriname and Colombia make providing timely trauma care challenging.

### Capacity

A total of 16% (9/57) raised ‘capacity’ as a potential barrier [36, 42–44, 52, 55, 60, 71, 72]. Four articles mentioned overcrowding with two highlighting the contrast between private and public care where the latter is more crowded. Three articles specifically outlined how high demand generally affects care, while others discussed the high demand and low provision for more specialist services, such as intensive care units in rural Bolivia and specialist polytrauma beds in Brazil.

### Public education

A total of 7% (4/57) mention lack of public education as a barrier to trauma care [52, 71, 72, 78]. One article highlights inappropriate use of services for issues that could be dealt with in primary care. Two articles discuss the lack of education around seeking timely care and attending follow-up. Two papers from Brazil highlight the issue of poor public education on traffic laws and road safety, alongside poor basic first aid. One paper describes how some patients use traditional healers for trauma care.

### Socio-cultural factors

A total of 23% (13/57) papers discuss ‘socio-cultural’ barriers [25, 31, 33, 34, 40, 41, 46, 53, 56, 58, 60, 62, 71]. The hierarchical nature of teams and poor communication between team members was raised in seven articles. Themes such as fear of seniors, inter-speciality conflicts and misunderstandings were common. Two articles raised the issue of healthcare workers and systems resisting change, such as reluctance to convert to electronic patient records. An article from Peru reported how a culture of Quality Improvement projects was being used to shame clinicians opposed to generate positive change. Two papers raised the problems associated with clinicians undertaking both private and public work, leading to concerns such as private work happening in scheduled quality improvement time.

## Risk of bias

**Fig. 4:**
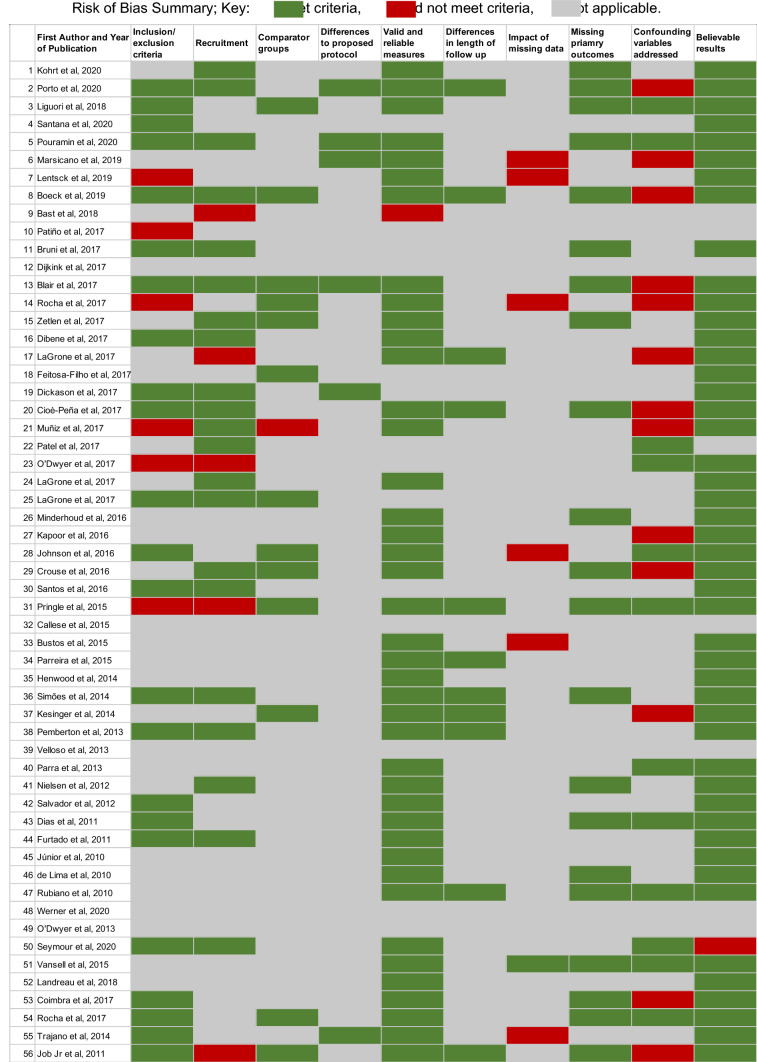
Risk of bias summary; key: green square—met criteria, red square—did not meet criteria, ash square—not applicable (colour figure online)

Fourteen articles [25, 29, 33, 47, 55, 56, 59, 62, 64, 66, 68, 69, 75, 79] were assessed against the RTI item bank for risk of bias. Five articles were reviews or reports [39, 44, 54, 76, 77], and therefore could not be evaluated. For most of the articles reviewed, criteria in the RTI item bank was not applicable, but the majority of articles were considered to use valid and reliable measures and to have plausible results (Fig. 4).

## Discussion

The results demonstrate the complex nature of trauma care and the multitude of factors which contribute. This systematic review highlights the different types of barriers which all contribute, from pre-hospital care and access to ambulance services to a lack of postgraduate training and dismissal of quality improvement projects. South and Central America comprise of mainly HIC and UMICs, with only four recorded as LMICs. Considering that the majority of articles cite lack of resources and finance as key barriers, it raises question of whether this is due to unequitable distribution rather than a true lack of capital.

Lack of training was the most frequently cited barrier. Access to specialist training and a commitment to continued professional development and quality improvement are important for providing high-quality healthcare, and are seen as a positive marker of a countries healthcare system [80, 81]. The poor availability and uptake of Emergency Medicine speciality training likely has a profound effect on the quality of care for trauma patients. Without more universal analysis of specialty training in the region, it is difficult to know if this is a general reflection of medical training or specific to Emergency Medicine. This may reflect one of the most straightforward barriers to improve, as it has been shown by Pringle et al. [63] that hugely beneficial effects from low-cost interventions such as simulation training courses make a significant difference to the quality of care provided.

On the theme of quality improvement, it appears that its importance is not always recognised, and overall there is a lack of commitment and networks in place to facilitate its delivery [8, 30, 35]. This is in part due to perceived lack of desire to change practise, and occasionally less of a drive for evidence-based medicine [35].

This links with the barriers highlighted in terms of lack of protocol and socio-cultural factors. This review demonstrated that standardised protocols and guidelines were often not implemented. Protocols for accurate and timely record-keeping were rare. The importance of good record-keeping to facilitate quality improvement is well-reported [82]**.** Further, there is a lack of protocols for triage, a vitally recognised aspect of trauma care. Quality improvement work and adherence to protocol require cohesive teamwork [83]; poor communication and imposed hierarchy were barriers highlighted across several articles. Finally, the lack of specialist training and transfer of staff to more lucrative specialities is widespread.

This review highlights the lack of research into the root cause of barriers, despite the profound morbidity and mortality associated with trauma. Most of the literature focussed on either single centres or regions, which may not reflect the economic status of large parts of South and Central America [22], and there is little available on the continent as a whole. Further, there is often a lack of distinction between pre-hospital and emergency and trauma care making it difficult to determine if barriers are specific to trauma or general management issues. The majority of the current research focusses on urban centres, and it is unclear to what extent the barriers are the shared or differ in comparison with rural South America.

## Limitations

The review was limited to English language only, whereas the predominant first languages of the region are Spanish and Portuguese. Fortunately, English translations were often available, and these were included where possible. Disparity in the data available for countries was apparent. While this review aims to encompass the whole of South America, many countries are considerably under-represented compared to those from which the bulk of the research is from, such as Brazil. We believe this provides further support for a Delphi Analysis.

## Conclusion

In South America, trauma is widespread and access to safe, timely, affordable and high-quality care is essential. This systematic review highlights the variety of barriers to provide high-quality trauma care, many of which interlink and appear to act in a synergistic fashion. It is of great importance that barriers to the provision of trauma care are recognised and addressed in order to address the associated mortality and morbidity. Many countries are under-represented in the literature, and the authors believe a Delphi analysis would best facilitate the pooling of expert knowledge and opinion for tackling complex barriers and considering how change can be implemented.

## Appendix

See Figs. 1, 2, 3 and 4. Tables 1 and 2.

**Table 1:** Inclusion and exclusion criteria used to identify relevant articles

	Inclusion criteria	Exclusion criteria
Language	English	Non-English
Study type	Qualitative or observational study, systematic review/evaluations	Case reviews/case control studies (*n* < 5), editorials, letters, conference abstracts
Topic	Trauma as per pre-defined definition	Other emergency medicine topics e.g. obstetrics, sepsis, myocardial infarction, stroke
Focus	Barriers to trauma care/services	Relating to rehabilitation, long-term care or non-medical/healthcare interventions
Area	Reference to/ based in South or Central America	No direct reference to South or Central America

**Table 2:** Summary table of all articles reviewed

	References	Paper title	Country	Aspect of ‘Trauma Care’ studied	Specific category of barriers identified
1	Kohrt et al. [56]	Establishing Context to Build Capacity: A Qualitative Study to Determine the Feasibility, Utility, and Acceptability of a Complex Trauma Training for Psychologists Working in Urban Migrant Communities in Northern Peru	Peru	Training	Training Financial Capacity Socio-cultural factors
2	Porto et al. [70]	Pattern of Maxillofacial Trauma and Associated Factors in Traffic Accident Victims	Brazil	Characteristics of Accident victims	Financial
3	Liguori et al. [20]	Basic Seismic Response Capability of Hospitals in Lima, Peru	Peru	Seismic response capability	Resources/equipment Protocol Transport/geographical Building/infrastructure
4	Santana et al. [21]	Factors that interfere in the quality of service to the critical patient	Brazil	Factors affecting care of the critical patient	Training Protocol Staff Equipment Capacity Building/infrastructure
5	Pouramin et al. [22]	Delays in hospital admissions in patients with fractures across 18 low-income and middle-income countries (INORMUS): a prospective observational study	Various—Venezuela	Causes of delays in hospital admissions	Resources Protocol Staff Financial Transport/geographical Public education
6	Marsicano et al. [57]	Epidemiology of Maxillofacial Trauma in a Prehospital Service in Brazil	Brazil	Maxillofacial trauma in pre-hospital setting	Training Protocol
7	Lentsck et al. [23]	Epidemiological overview—18 years of ICU hospitalization due to trauma in Brazil	Brazil	ICU trauma admissions	Resources
8	Boeck et al., 2019 [55]	The development and implementation of a layperson trauma first responder course in La Paz, Bolivia: A pilot study	Bolivia	Training development	Training Protocol
9	Bast et al. [24]	Challenges to Prehospital Care in Honduras	Honduras	Pre-hospital care challenges	Resources Protocol Staff Financial Transport/geographical Capacity Public education
10	Patiño et al., 2017 [52]	Characteristics of emergency medicine residency programs in Colombia	Colombia	Teaching/Training	Training Staff Financial
11	Bruni et al. [25]	A qualitative assessment of trauma care at Georgetown Public Hospital Corporation in Guyana	Guyana	Assessment of hospital trauma care	Resources Training Socio-cultural factors
12	Dijkink et al. [26]	Trauma systems around the world: A systematic overview	Brazil	Assessment of trauma systems	Resources Protocol
13	Blair et al. [27]	Assessment of Surgical and Trauma Capacity in Potosi, Bolivia	Bolivia	Surgical and trauma capacity	Resources/equipment Training Protocol Staff Transport/geographical Capacity
14	Rocha et al. [28]	Access to emergency care services: a transversal ecological study about Brazilian emergency health care network	Brazil	Emergency service access	Resources Transport/geographical
15	Zetlen et al. [8]	Status of trauma quality improvement programs in the Americas: a survey of trauma care providers	Various—Bolivia, Guatemala, Nicaragua, Brazil, Colombia, Costa Rica, Ecuador, Panama, Paraguay, Peru, Venezuela, Argentina, Chile, Uruguay	Quality improvement	Resources Training Protocol Staff
16	Dibene et al. [29]	Optimizing the location of ambulances in Tijuana, Mexico	Mexico	Emergency response/ Ambulances	Resources Transport/geographical
17	LaGrone et al. [30]	Surgeons' and Trauma Care Physicians' perception of the impact of the globalization of medical education on quality of care in Lima, Peru	Peru	Medical education	Resources Training Protocol Staff Financial
18	Feitosa-Filho et al. [58]	Characteristics of training and motivation of physicians working in emergency medicine	Brazil	Training and Motivation	Training Socio-cultural factors
19	Dickason et al. [31]	Primary trauma care curriculum: A qualitative analysis of impediments to improvement	El Salvador	Trauma curriculum	Training Protocol Staff 20Equipment Financial Socio-cultural factors
20	Cioè-Peña et al. [32]	Development and implementation of a hospital-based trauma response system in an urban hospital in San Salvador, El Salvador	El Salvador	Trauma response systems—development	Resources Training Protocol Staff Transport/geographical
21	Muñiz et al. [59]	Preparing Global Trauma Nurses for Leadership Roles in Global Trauma Systems	Various—Colombia, Uruguay	Training/global leadership	Training
22	Patel et al. [33]	Qualitative evaluation of trauma delays in road traffic injury patients in Maringa, Brazil	Brazil	Delays of road traffic injury patients	Resources Protocol Staff Transport/geographical Public education Socio-cultural factors
23	O'Dwyer et al. [34]	The process of implementation of emergency care units in Brazil	Brazil	Implantation of emergency care units	Resources Training 24Protocol St2aff Financial Socio-cultural factors
24	LaGrone et al. [35]	Mixed-Methods Assessment of Trauma and Acute Care Surgical Quality Improvement Programs in Peru	Peru	Quality improvement	Resources/equipment Training Protocol Staff Socio-cultural factors
25	LaGrone et al. [53]	Surgery and trauma care providers' perception of the impact of dual-practice employment on quality of care provided in an Andean country	Peru	Care quality	Training Staff Socio-cultural factors
26	Minderhoud et al. [73]	Epidemiology and aetiology of childhood ocular trauma in the Republic of Suriname	Suriname	Epidemiology and aetiology	Transport/geographical Public education
27	Kapoor et al. [60]	Regional scale-up of an Emergency Triage Assessment and Treatment (ETAT) training programme from a referral hospital to primary care health centres in Guatemala	Guatemala	Triage and treatment training program	Training Protocol Transport/geographical Socio-cultural factors
28	Johnson et al. [36]	Emergency department of a rural hospital in Ecuador	Ecuador	Emergency department in a rural hospital	Resources Training
29	Crouse et al., 2016 [61]	Quality and Effectiveness of a Pediatric Triage Training Program in a Guatemalan Public Hospital	Guatemala	Paediatric triage training program	Training Protocol
30	Santos et al. [62]	Elder-friendly emergency services in Brazil: necessary conditions for care	Brazil	Emergency service analysis	Training Protocol Socio-cultural factors
31	Pringle et al. [63]	A short trauma course for physicians in a resource-limited setting: Is low-cost simulation effective?	Nicaragua	Trauma simulation training	Training
32	Callese et al. [71]	Trauma system development in low- and middle-income countries: A review	Various—Guatemala	System development	Financial Socio-cultural factors
33	Bustos et al. [37]	Emergency department characteristics and capabilities in Bogota, Colombia	Colombia	Emergency department—review	Resources Training
34	Parreira et al. [68]	Implementation of the trauma registry as a tool for quality improvement in trauma care in a Brazilian hospital: the first 12 months	Brazil	Quality improvement	Protocol Financial
35	Henwood et al. [38]	Characterizing the limited use of point-of-care ultrasound in Colombian emergency medicine residencies	Colombia	Use of USS in emergency medicine	Resources Training Financial Socio-cultural factors
36	Simões et al. [64]	Trauma leagues: an alternative way to teach trauma surgery to medical students	Brazil	Trauma surgery training	Training
37	Kesinger et al. [39]	Improving trauma care in low- and middle-income countries by implementing a standardized trauma protocol	Colombia	Trauma protocol	Resources Protocol Financial
38	Pemberton et al. [40]	Evaluating the long-term impact of the Trauma Team Training course in Guyana: An explanatory mixed-methods approach	Guyana	Trauma training	Resources Training Socio-cultural factors
39	Velloso et al. [41]	Configurations of power relations in the Brazilian emergency care system: Analysing a context of visible practices	Brazil	System review	Resources Socio-cultural factors
40	Parra et al. [42]	International trauma teleconference: evaluating trauma care and facilitating quality improvement	Various—Colombia, Ecuador	Evaluation of trauma care and quality improvement	Resources/equipment Training Protocol
41	Nielsen et al. [43]	Assessment of the status of prehospital care in 13 low- and middle-income countries	Various—Peru, Brazil, Ecuador, Colombia, Panama	Pre-hospital care	Resources Training Protocol Financial Transport/geographical
42	Salvador et al. [65]	Profile of Brazilian dissertations and theses on trauma: A documentary research	Brazil	Profile of trauma research	Training
43	Dias et al. [66]	Entrapped victims in motor vehicle collisions: Characteristics and prehospital care in the city of Sao Paulo, Brazil	Brazil	Prehospital care	Training Protocol Capacity Public education
44	Furtado et al. [67]	Clinic-epidemiological analysis of an Otorhinolaryngology emergency unit care in a tertiary hospital	Brazil	Tertiary hospital emergency unit	Training Protocol Financial Capacity Public education
45	Júnior et al. [44]	Emergency medical coordination using a web platform: A pilot study	Brazil	System coordination and management	Resources Training Protocol Capacity
46	de Lima et al., 2010 [45]	An analysis of prehospital care for victims of accidents and violence in Recife, Brazil	Brazil	Prehospital care	Resources/equipment Training Protocol Capacity
47	Rubiano et al. [46]	Trauma care training for national police nurses in Colombia	Colombia	Trauma care training	Resources Training Transport/geographical Socio-cultural factors
48	Werner et al. [72]	Cost-effectiveness of emergency care interventions in low and middle income countries: a systematic review	Various—Brazil, Colombia, Paraguay	Cost-effectiveness of interventions	Financial
49	O'Dwyer et al. [47]	The current scenario of emergency care policies in Brazil	Brazil	Emergency care policies	Resources/equipment Training Protocol Staff Capacity Building/infrastructure
50	Seymour et al., 2020 [48]	Efficacy of Distance-Based EMS Education in a Low-Resource Country	Belize	Distance education	Resources Training
51	Vansell et al. [49]	Anaesthesia, surgery, obstetrics, and emergency care in Guyana	Guyana	Emergency care	Resources/equipment Training Staff Financial Transport/geographical Building/infrastructure
52	Landreau et al. [50]	Helicopter Emergency Medical Services in Buenos Aires: An Operational Overview	Argentina	Air emergency services	Resources Transport/geographical
53	Coimbra et al., 2017 [51]	Analysis of the availability of the resources necessary for urgent and emergency healthcare in Sao Paulo between 2009–2013	Brazil	Availability of resources	Resources Staff
54	Rocha et al. [74]	Addressing geographic access barriers to emergency care services: a national ecologic study of hospitals in Brazil	Brazil	Access to care	Transport/geographical
55	Trajano et al. [69]	Epidemiology of in-hospital trauma deaths in a Brazilian university hospital	Brazil	Epidemiology of trauma deaths	Protocol Transport/geographical
56	Job Jr et al. [54]	Evaluation of the effectiveness of systematized training of advanced trauma life support protocol in the interpretation of cervical spine and chest radiographs in three different emergency services	Brazil	Training	Training Protocol Staff

## References

[1] Physical Trauma [Internet]. National Institute of General Medical Sciences. 2020 [cited 2020 Nov 2]. https://www.nigms.nih.gov/education/fact-sheets/Pages/physical-trauma.aspx

[2] Padilla Rojas LG, López Cervantes RE, Pérez Atanasio JM, Sánchez MM, Gómez Acevedo JM, Kojima KE (2019). Latin America trauma systems—Mexico and Brazil. OTA Int.

[3] Almanza-Cruz S, Rea-Field G. A model system for emergency medicine for Mexico City. Gac Med Mex. 1990 2103552

[4] Smith GS, Barss P (1991). Unintentional injuries in developing countries: the epidemiology of a neglected problem. Epidemiol Rev.

[5] Nilsen P, Hudson D, Lindqvist K (2006). Economic analysis of injury prevention–applying results and methodologies from cost-of-injury studies. Int J Inj Contr Saf Promot.

[6] Kotagal M, Agarwal-Harding KJ, Mock C, Quansah R, Arreola-Risa C, Meara JG (2014). Health and economic benefits of improved injury prevention and trauma care worldwide. PLoS ONE.

[7] WHO. World Bank country and Lending Groups [Internet]. 2020 [cited 2020 Nov 2]. https://datahelpdesk.worldbank.org/knowledgebase/articles/906519-world-bank-country-and-lending-groups

[8] Mock C, Joshipura M, Arreola-Risa C, Quansah R (2012). An estimate of the number of lives that could be saved through improvements in trauma care globally. World J Surg.

[9] Zetlen HL, LaGrone LN, Foianini JE, Egoavil EH, Sproviero J, Rivera FV (2017). Status of trauma quality improvement programs in the Americas: a survey of trauma care providers. J Surg Res.

[10] ECLAC. Poverty in Latin America Remained Steady in 2017, but Extreme Poverty Increased to the Highest Level since 2008, while Inequality has Fallen Notably since 2000. 2020.

[11] Lopez JH, Perry G. Inequality in Latin America: determinants and consequences [Internet]. Policy Research Working Papers. The World Bank; 2008. 41 p. 10.1596/1813-9450-4504

[12] Aboutanos MB, Mora F, Rodas E, Salamea J, Parra MO, Salgado E (2010). Ratification of IATSIC/WHO’s guidelines for essential trauma care assessment in the South American Region. World J Surg.

[13] Kanavos P, Parkin GC, Kamphuis B, Gill J. Latin America Healthcare System Overview: a comparative analysis of fiscal space in healthcare. London Sch Econ Polit Sci. 2019;(August):16.

[14] Jones MD, Kalamchi LD, Schlinkert AB, Chapple KM, Jacobs JV, Bogert JN (2020). Are all trauma centers created equal? Level 1 to level 1 trauma center patient transfers in the setting of rapid trauma center proliferation. J Trauma Acute Care Surg.

[15] American Trauma Society. Trauma Center Levels Explained [Internet]. [cited 2021 Jun 23]. https://www.amtrauma.org/page/traumalevels

[16] Carpenter B, Levine L, Niacaris T, Suzuki S (2017). Transitioning from a level II to level I trauma center increases resident patient exposure. J Foot Ankle Surg.

[17] WHO. Injuries and violence: the facts [Internet]. 2010 [cited 2020 Oct 25]. https://www.who.int/violence_injury_prevention/key_facts/en/

[18] Haagsma JA, Graetz N, Bolliger I, Naghavi M, Higashi H, Mullany EC (2016). The global burden of injury: incidence, mortality, disability-adjusted life years and time trends from the Global Burden of Disease study 2013. Inj Prev.

[19] Gosselin RA, Spiegel DA, Coughlin R, Zirkle LG (2009). Injuries: the neglected burden in developing countries. Bull World Health Organ.

[20] WHO. Global health estimates summary tables: projection of deaths by cause, age and sex, by world bank income group [Internet]. 2018 [cited 2020 Sep 11]. Available from: https://www.who.int/healthinfo/global_burden_disease/projections/en/

[21] Whitaker J, Nepogodiev D, Leather A, Davies J (2020). Assessing barriers to quality trauma care in low and middle-income countries: a Delphi study. Injury.

[22] Haghparast-Bidgoli H, Hasselberg M, Khankeh H, Khorasani-Zavareh D, Johansson E. Barriers and facilitators to provide effective pre-hospital trauma care for road traffic injury victims in Iran: a grounded theory approach. BMC emergency medicine. 2010 Dec;10(1):1–1. 10.1186/1471-227X-10-20 PMC2992044 21059243

[23] Kironji AG, Hodkinson P, De Ramirez SS, Anest T, Wallis L, Razzak J, Jenson A, Hansoti B. Identifying barriers for out of hospital emergency care in low and low-middle income countries: a systematic review. BMC health services research. 2018 Dec;18(1):1-20. 10.1186/s12913-018-3091-0 PMC5907770 29673360

[24] Kruk ME, Gage AD, Arsenault C, Jordan K, Leslie HH, Roder-DeWan S (2018). High-quality health systems in the sustainable development goals era: time for a revolution. Lancet Glob Heal.

[25] Liguori N, Tarque N, Bambaren C, Santa-Cruz S, Palomino J, Laterza M (2019). Basic seismic response capability of hospitals in Lima, Peru. Disaster Med Public Health Prep.

[26] Dibene JC, Maldonado Y, Vera C, de Oliveira M, Trujillo L, Schütze O (2017). Optimizing the location of ambulances in Tijuana, Mexico. Comput Biol Med.

[27] LaGrone LN, Isquith-Dicker LN, Egoavil EH, Castro MJ, Allagual A, Revoredo F, Mock CN (2017) Surgeons’ and trauma care physicians’ perception of the impact of the globalization of medical education on quality of care in Lima, Peru. JAMA Surg 152(3):251–256 10.1001/jamasurg.2016.4073 27893012

[28] Dickason RM, Cioè-Peña E, Chisolm-Straker M (2017). Primary trauma care curriculum: a qualitative analysis of impediments to improvement. Trauma (United Kingdom).

[29] Cioè-Peña EC, Granados JC, Wrightsmith LL, Henriquez-Vigil AL, Moresky RT (2017) Development and implementation of a hospital-based trauma response system in an urban hospital in San Salvador, El Salvador. Trauma (United Kingdom)

[30] Patel A, Vissoci JRN, Hocker M, Molina E, Gil NM, Staton C (2017). Qualitative evaluation of trauma delays in road traffic injury patients in Maringá. Brazil. BMC Health Serv Res.

[31] O'Dwyer G, Konder MT, Reciputti LP, Lopes MG, Agostinho DF, Alves GF (2017) The process of implementation of emergency care units in Brazil. Revista de saude publica 51. 10.11606/S1518-8787.2017051000072 PMC5718113 29236876

[32] LaGrone LN, Fuhs AK, Egoavil EH, Rodriguez Castro MJA, Valderrama R, Isquith-Dicker LN (2017). Mixed-methods assessment of trauma and acute care surgical quality improvement programs in Peru. World J Surg.

[33] Johnson T, Gaus D, Herrera D (2016). Emergency department of a rural hospital in Ecuador. West J Emerg Med.

[34] Bustos Y, Castro J, Wen LS, Sullivan AF, Chen DK, Camargo CA (2015) Emergency department characteristics and capabilities in Bogotá, Colombia. Int J Emerg Med 8(1):1–8 10.1186/s12245-015-0079-y PMC4529430 26253755

[35] Henwood PC, Beversluis D, Genthon AA, Wilson CN, Norwood B, Silva D, Foran M, Romero MG, Martinez YB, Vargas LE, Ocampo AC (2014) Characterizing the limited use of point-of-care ultrasound in Colombian emergency medicine residencies. Int J Emerg Med 7(1):1–7 10.1186/1865-1380-7-7 PMC3922404 24499650

[36] da Santana RS, Dias EMDS, de Oliveira GS, de Batista CA, Frota CA, da Silva RK (2020). Factors that interfer in the quality of service to the critical patient. Acta Sci Heal Sci.

[37] Kesinger MR, Puyana JC, Rubiano AM (2014). Improving trauma care in low- and middle-income countries by implementing a standardized trauma protocol. World J Surg.

[38] Pemberton J, Rambaran M, Cameron BH (2013). Evaluating the long-term impact of the Trauma Team Training course in Guyana: an explanatory mixed-methods approach. Am J Surg.

[39] Velloso I, Ceci C, Alves M (2013). Configurations of power relations in the Brazilian emergency care system: analyzing a context of visible practices. Nurs Inq.

[40] Parra MW, Castillo RC, Rodas EB, Suarez-Becerra JM, Puentes-Manosalva FE, Wendt LM (2013). International trauma teleconference: evaluating trauma care and facilitating quality improvement. Telemed e-Health.

[41] Nielsen K, Mock C, Joshipura M, Rubiano AM, Zakariah A, Rivara F (2012). Assessment of the status of prehospital care in 13 low-and middle-income countries. Prehospital Emerg Care.

[42] Adolfi Júnior MS, Pallini FM, Pessotti H, Wolf CM, Patelli HT, Capeli RD et al (2010) Emergency medical coordination using a web platform: a pilot study. Rev Saude Publica 10.1590/s0034-89102010000600011 21107504

[43] de Lima MLC, de Souza ER, Deslandes SF, Kelly A, de Santana Cabral AP (2010). An analysis of prehospital care for victims of accidents and violence in Recife.

[44] O’Dwyer G, Konder MT, Machado CV, Alves CP, Alves RP (2013) The current scenario of emergency care policies in Brazil. BMC Health Serv Res 10.1186/1472-6963-13-70 PMC3598552 23425342

[45] Seymour L, Montejo R, Weston BW (2020) Efficacy of Distance-Based EMS Education in a Low-Resource Country. Environments 4:5 33091286

[46] Vansell HJ, Schlesinger JJ, Harvey A, Rohde JP, Persaud S, McQueen KA (2015). Anaesthesia, surgery, obstetrics, and emergency care in Guyana. J Epidemiol Glob Health.

[47] Pouramin P, Li CS, Busse JW, Sprague S, Devereaux PJ, Jagnoor J (2020). Delays in hospital admissions in patients with fractures across 18 low-income and middle-income countries (INORMUS): a prospective observational study. Lancet Glob Heal.

[48] Coimbra SH, Camanho EDL, Heringer LC, Botelho RV, Vasconcellos C (2017). Analysis of the availability of the resources necessary for urgent and emergency healthcare in São Paulo between 2009–2013. Rev Assoc Med Bras.

[49] Rubiano AM, Sánchez ÁI, Guyette F, Puyana JC (2010). Trauma care training for national police nurses in Colombia. Prehospital Emerg Care.

[50] Landreau F, Valcarcel O, Noir J, Pernía G, Orzábal ML, Martínez S (2018). Helicopter emergency medical services in Buenos Aires: an operational overview. Air Med J.

[51] Lentsck MH, Sato APS, de Mathias TAF (2019). Epidemiological overview - 18 years of ICU hospitalization due to trauma in Brazil. Rev Saude Publica.

[52] Bast HE, Jenkins JL (2018). Challenges to prehospital care in Honduras. Prehosp Disaster Med.

[53] Bruni A, Kazmi K, Bailey K, Rambaran N, Rambaran M, Cameron B et al (2017) A qualitative assessment of trauma care at Georgetown Public Hospital Corporation in Guyana. West Indian Med J

[54] Dijkink S, Nederpelt CJ, Krijnen P, Velmahos GC, Schipper IB (2017). Trauma systems around the world: a systematic overview. J Trauma Acute Care Surg.

[55] Blair KJ, Boeck MA, Barrientos JL, López JL, Helenowski IB, Nwomeh BC, Shapiro MB, Swaroop M (2017) Assessment of surgical and trauma capacity in Potosí, Bolivia. Ann Global Health 83(2):262–273 10.1016/j.aogh.2017.04.002 28619401

[56] Rocha TAH, da Silva NC, Amaral PV, Barbosa ACQ, Rocha JVM, Alvares V (2017). Access to emergency care services: a transversal ecological study about Brazilian emergency health care network. Public Health.

[57] Patiño A, Alcalde V, Gutierrez C, Romero MG, Carrillo AM, Vargas LE (2017). Characteristics of emergency medicine residency programs in Colombia. West J Emerg Med.

[58] LaGrone LN, Isquith-Dicker LN, Huaman Egoavil E, Herrera-Matta JJ, Fuhs AK, Ortega Checa D (2017). Surgery and trauma care providers’ perception of the impact of dual-practice employment on quality of care provided in an Andean country. Br J Surg.

[59] Job PM, Von Bahten LC, de Oliveira-Junior N (2011). Evaluation of the effectiveness of systematized training of advanced trauma life support protocol in the interpretation of cervical spine and chest radiographs in three different emergency services. J Trauma.

[60] Kohrt BK, Murray MP, Cabel SL (2020). Establishing context to build capacity: a qualitative study to determine the feasibility, utility, and acceptability of a complex trauma training for psychologists working in urban migrant communities in Northern Peru. Community Ment Health J.

[61] Marsicano JA, Cavalleri NZ, Cordeiro DM, Mori GG, da Silveira JL, do Prado RL (2019) Epidemiology of maxillofacial trauma in a prehospital service in Brazil. J Trauma Nurs 26(6):323–327. 10.1097/JTN.0000000000000470 31714493

[62] Boeck MA, Callese TE, Nelson SK, Schuetz SJ, Bazan CF, Laguna JM, Shapiro MB, Issa NM, Swaroop M (2018) The development and implementation of a layperson trauma first responder course in La Paz, Bolivia: A pilot study. Injury 49(5):885–896 10.1016/j.injury.2017.11.022 29198373

[63] Feitosa-Filho GS, Kirschbaum M, Neves YCS, Loureiro BMC, De Castro Lima VAC, Calazans RM (2017). Characteristics of training and motivation of physicians working in emergency medicine. Rev Assoc Med Bras.

[64] Muñiz SA, Lang RW, Falcon L, Garces-King J, Willard S, Peck GL (2017). Preparing global trauma nurses for leadership roles in global trauma systems. J Trauma Nurs.

[65] Kapoor R, Sandoval MA, Avendaño L, Cruz AT, Soto MA, Camp EA (2016). Regional scale-up of an Emergency Triage Assessment and Treatment (ETAT) training programme from a referral hospital to primary care health centres in Guatemala. Emerg Med J.

[66] Crouse HL, Vaides H, Torres F, Ishigami EM, Walsh MT, Soto MA (2016). Quality and effectiveness of a pediatric triage training program in a Guatemalan Public Hospital. Pediatr Emerg Care.

[67] Dos Santos MT, da Lima MADS, Zucatti PB (2016). Elder-friendly emergency services in Brazil: necessary conditions for care. Rev Esc Enferm USP.

[68] Pringle K, Mackey JM, Modi P, Janeway H, Romero T, Meynard F (2015). A short trauma course for physicians in a resource-limited setting: Is low-cost simulation effective?. Injury.

[69] Simões RL, Bermudes FAM, Andrade HS, Barcelos FM, Rossoni BP, Miguel GPS (2014). Trauma leagues: an alternative way to teach trauma surgery to medical students. Rev Col Bras Cir.

[70] de Salvador PTCO, Alves KYA, Martins CCF, Santos VEP, Tourinho FSV (2012). Perfil das dissertações e teses brasileiras acerca do trauma: uma pesquisa documental TT - Profile of brazilian dissertations and theses on trauma: a documentary research. Rev Col Bras Cir.

[71] Dias ARN, Abib S de CV, Poli-de-Figueiredo LF, Perfeito JAJ (2011) Entrapped victims in motor vehicle collisions: characteristics and prehospital care in the city of São Paulo, Brazil. Clinics 10.1590/S1807-59322011000100005 PMC3044585 21437431

[72] Furtado PL, Nakanishi M, Rezende GL, Granjeiro RC, de Oliveira TS (2011). Clinic-epidemiological analysis of an Otorhinolaryngology emergency unit care in a tertiary hospital. Braz J Otorhinolaryngol.

[73] Parreira JG, Campos TD, Perlingeiro JA, Soldá SC, Assef JC, Gonçalves AC, ZUFFO B, Floriano CG, Oliveira EH, Oliveira RV, Oliveira AL (2015) Implementation of the trauma registry as a tool for quality improvement in trauma care in a Brazilian hospital: the first 12 months. Revista do Colégio Brasileiro de Cirurgiões 42:265–72. 10.1590/0100-69912015004012 26517803

[74] Trajano AD, Pereira BM, Fraga GP (2014) Epidemiology of in-hospital trauma deaths in a Brazilian university hospital. BMC Emerg Med 14(1):1–9 10.1186/1471-227X-14-22 PMC4220277 25361609

[75] Porto DE, do Araújo JMN, Júnior CL, de Andrade ESS (2020). Pattern of maxillofacial trauma and associated factors in traffic accident victims. J Craniofac Surg.

[76] Callese TE, Richards CT, Shaw P, Schuetz SJ, Paladino L, Issa N (2015). Trauma system development in low- and middle-income countries: a review. J Surg Res.

[77] Werner K, Risko N, Burkholder T, Munge K, Wallis L, Reynolds T (2020). Cost–effectiveness of emergency care interventions in low and middle-income countries: a systematic review. Bull World Health Organ.

[78] Minderhoud J, van Nispen RMA, Heijthuijsen AAAM, Beunders VAA, Bueno de Mesquita-Voigt AMT, Moll AC (2016). Epidemiology and aetiology of childhood ocular trauma in the Republic of Suriname. Acta Ophthalmol.

[79] Rocha TAH, Da Silva NC, Amaral PV, Barbosa ACQ, Rocha JVM, Alvares V (2017). Addressing geographic access barriers to emergency care services: A national ecologic study of hospitals in Brazil. Int J Equity Health.

[80] Quality improvement training for healthcare professionals. Heal Found [Internet]. 2012;(August). https://www.health.org.uk/sites/default/files/QualityImprovementTrainingForHealthcareProfessionals.pdf

[81] Sherbino J, Bandiera G, Doyle K, Frank JR, Holroyd BR, Jones G (2020). The competency-based medical education evolution of Canadian emergency medicine specialist training. CJEM.

[82] Glen P, Earl N, Gooding F, Lucas E, Sangha N, Ramcharitar S (2015). Simple interventions can greatly improve clinical documentation: a quality improvement project of record keeping on the surgical wards at a district general hospital. BMJ Qual Improv Rep.

[83] Rowland P, Lising D, Sinclair L, Baker GR (2018). Team dynamics within quality improvement teams: a scoping review. Int J Quality Health Care.

